# Data warehousing in healthcare: enhancing resource allocation and decision-making in Botswana

**DOI:** 10.1093/oodh/oqag006

**Published:** 2026-03-09

**Authors:** Alton Mabina, Gabofetswe Malema, Cleverence Kombe

**Affiliations:** Department of Computer Science, University of Botswana, Gaborone, Botswana; Department of Computer Science, University of Botswana, Gaborone, Botswana; Department of Computer Science, University of Botswana, Gaborone, Botswana

**Keywords:** data warehousing, healthcare, resource allocation, decision support systems

## Abstract

Healthcare systems in low- and middle-income countries (LMICs) face persistent challenges in resource allocation due to fragmented health information systems and limited decision support capacity. In Botswana, despite significant investments in platforms such as District Health Information Software, Integrated Patient Management System, and Patient Information Management System, these systems remain siloed, constraining real-time analytics and equitable planning. Existing literature has primarily emphasized isolated platforms or high-level policy discussions, leaving a critical gap in empirically grounded, methodologically rigorous frameworks for integrating disparate datasets into unified, decision-oriented architectures. This study addresses that gap by designing and evaluating a Kimball-based dimensional data warehousing framework tailored to Botswana’s healthcare ecosystem. Guided by the Preferred Reporting Items for Systematic Reviews and Meta-Analyses framework, a systematic review of 72 peer-reviewed articles published between 2021 and 2025 was conducted, focusing on modeling approaches, interoperability practices, and operational outcomes. Results reveal a clear preference for Kimball’s bottom-up dimensional modeling, particularly star schemas, due to their incremental deployment, user-centered design, and suitability for resource-constrained environments. Thematic synthesis highlights medicine stockouts, staffing mismatches, and budget execution delays as recurring bottlenecks, with evidence that robust ETL pipelines and source-to-target mapping significantly improve data reliability and decision-making. Findings demonstrate that a localized Kimball framework can transform fragmented health data into actionable intelligence, enabling real-time visibility of staffing, inventory, and financial flows. The study concludes that such an approach not only strengthens efficiency and equity in Botswana’s healthcare system but also offers a transferable model for other LMICs seeking to align technical innovation with governance realities and operational needs.

## Introduction

This study is significant as it addresses a critical bottleneck in healthcare systems within developing countries the lack of integrated, data-driven decision support for effective resource allocation. By proposing a tailored data warehousing solution built on the Kimball methodology, the study empowers healthcare managers in Botswana with real-time insights, predictive analytics, and decentralized autonomy, thereby promoting efficiency, transparency, and equity in service delivery. Its importance lies not only in the technical innovation but also in its context-specific application, filling a notable gap in both literature and practice. Similar studies have emerged across Africa and Asia such as Mataba and Chibaro (2024) in Zimbabwe, who applied the Inmon model to enhance hospital supply chains, and AbuKhousa et al. (2014) in Nigeria, who employed a Kimball approach for centralized patient care reporting in teaching hospitals. These parallel findings reinforce the relevance and replicability of this study’s approach, making it a valuable contribution to global health informatics strategies aimed at transforming data into actionable health policy in resource-limited settings (Seitio-Kgokgwe et al. 2015, Mabina et al. 2025a).

Seitio-Kgokgwe et al. (2015) mentioned that Botswana has made significant investments in digital health through national platforms such as District Health Information Software (DHIS2), the Integrated Patient Management System (IPMS), and disease-specific electronic registries, reflecting a strong policy commitment to evidence-based healthcare planning. However, these systems primarily support data capture and reporting and remain fragmented, limiting their ability to provide integrated, real-time decision support for strategic resource allocation. As a result, healthcare managers continue to rely on retrospective and manually aggregated reports, constraining responsiveness and equity, particularly across geographically dispersed and resource-variable settings. This study responds to this gap by proposing a Kimball-based data warehousing framework that integrates existing health information systems into a unified analytical layer, enabling timely insights, predictive analysis, and decentralized decision-making aligned with Botswana’s digital health and health system strengthening priorities.

A critical gap in the existing literature on healthcare information systems in Botswana and comparable low-resource settings lies in the absence of a contextually grounded, methodologically rigorous data warehousing framework explicitly designed to support equitable healthcare resource allocation. Prior studies have largely focused on isolated health information platforms or high-level policy discussions, offering limited empirical guidance on how fragmented national systems such as IPMS, DHIS, and Patient Information Management System (PIMS) can be systematically integrated into a unified analytical architecture (Bagherian and Sattari 2022). Moreover, the literature reveals a lack of user-centered, decision-oriented frameworks that translate routinely collected health data into actionable intelligence for real-time planning and operational management. Methodologically, bottom-up dimensional modelling approaches, particularly the Kimball methodology, remain underexplored within Botswana’s digital health research, despite their suitability for incremental implementation in resource-constrained environments (Mabina et al. 2025b). This gap underscores the need for an empirically informed, implementation-ready data warehousing framework that aligns technical design choices with Botswana’s institutional, infrastructural, and governance realities, an omission this study explicitly addresses while contributing a transferable model for other developing healthcare systems.

### Research gap

Despite significant investments in digital health platforms such as DHIS2, IPMS, and PIMS, existing research in Botswana and comparable low-resource settings remains insufficient in providing empirically grounded, methodologically rigorous frameworks for integrating fragmented health information systems into unified, decision-oriented architectures (Ndlovu et al. 2025). Prior studies have largely emphasized isolated systems or high-level policy discussions, resulting in a lack of clarity and consensus on how dimensional data warehousing can practically address persistent bottlenecks such as medicine stockouts, staffing mismatches, and budget execution delays. Methodological controversies persist between Kimball and Inmon approaches, with limited comparative evidence on their suitability for resource-constrained environments. Furthermore, previous research often neglects user-centered design and fails to resolve real-world challenges of interoperability, metadata governance, and decentralized decision support. This leaves a critical gap in both knowledge and practice: the absence of a contextually tailored, empirically validated data warehousing framework that aligns technical design with governance realities and operational needs in Botswana’s healthcare system (Mabina et al. 2025a).

### Research questions

- **RQ1:** Which dimensional modeling approaches Kimball, Inmon, hybrid are most frequently used and why in Low- and Middle-Income Countries health settings?
- **RQ2:** What technical and governance practices support reliable ETL and interoperability across DHIS2, IPMS, and PIMS?
- **RQ3:** What evidence exists that data-warehousing interventions improve operational outcomes?

### Research objective(s)

The goal of this study was to design and evaluate a Kimball-based dimensional data warehousing framework tailored to Botswana’s healthcare ecosystem, with the explicit aim of transforming fragmented health information systems into a unified analytical architecture for fair resource allocation. In doing so, the study provides policy makers and decision makers with a practical roadmap for integrating disparate datasets into a coherent, decision-oriented framework, thereby filling a critical knowledge gap in how data warehousing can be applied to strengthen healthcare planning in resource-constrained settings.

### Significance of the study

The significance of this study lies in its practical response to the persistent challenge of inefficient healthcare resource allocation in Botswana’s low-resource settings. By developing a localized, Kimball-based data warehousing solution, the research not only enhances real-time decision-making and data visibility but also empowers district health managers with analytical tools to respond more effectively to local healthcare needs. This intervention bridges the gap between fragmented health data systems and strategic planning, offering a scalable model for other developing nations facing similar systemic constraints (Nassoura 2024, Lyu et al. 2025).

This study is critical for policy makers because it provides an empirically grounded, context-specific framework that transforms fragmented health information systems into a unified, decision-oriented architecture. By adopting Kimball’s dimensional modeling and robust ETL pipelines, the framework enables real-time visibility of medicine stock levels, staffing resources, and budget execution, directly addressing persistent bottlenecks in Botswana’s healthcare system. This means moving beyond retrospective reporting toward proactive, evidence-based planning that supports equitable resource allocation across districts. The integration of explainable artificial intelligence (AI) forecasting and ethical guardrails further ensures transparency, accountability, and trust in decision support, aligning technical innovation with governance priorities. The study equips policy makers with actionable intelligence to strengthen efficiency, equity, and responsiveness in national health strategies (Casanova et al. 2025).

## Methods

The Preferred Reporting Items for Systematic Reviews and Meta-Analyses (PRISMA) framework guided the process through four key phases: identification, screening, eligibility, and inclusion. Initially, records were identified through comprehensive database searches. Duplicates were then removed, and the remaining records underwent screening based on titles and abstracts (Page et al. 2021). Subsequently, full-text articles were assessed for eligibility against predefined inclusion and exclusion criteria. Studies meeting all criteria were included in the qualitative synthesis. This structured approach ensured transparency and rigor in the selection process, enhancing the reliability of the review’s findings.

### Search strategy

Databases to perform the literature search included PubMed, IEEE Xplore, Scopus, Web of Science, and Google Scholar. Other sources involved citations contained in the articles retrieved, as well as pertinent gray literature identified through searches of institutional repositories and websites of healthcare organizations. All databases were searched from 2021 up to 2025 to be as comprehensive as possible, and the reference lists of all included studies were also checked manually for any additional relevant studies. A search strategy for this review was developed in conjunction with healthcare informatics and systematic review experts.

Search strategy utilized keywords and controlled vocabulary terms comprised of various combinations of the following: “data warehousing,” “healthcare,” “resource allocation,” “decision-making,” “analytics,” “database systems,” “health information systems.” Filters were applied for publication date, 2021–25 and language: English, and Boolean operators were used: AND, OR.

### Selection process

A detailed two-step screening process is followed in the selection. At first, two reviewers screened the titles and abstracts of all retrieved records to identify potentially relevant studies. The second step involved screening the full text of the shortlisted articles for eligibility mostly their methodology. Any differences between reviewers were either discussed or referred to a third reviewer. The screening and selection process was facilitated using Rayyan software.

### Critical appraisal

The critical appraisal of this research reveals a methodologically sound and contextually relevant investigation into the role of data warehousing in enhancing healthcare resource allocation. Utilizing the PRISMA framework, the study systematically reviewed 72 articles published within the last 5 years, ensuring contemporary relevance and academic rigor. The inclusion and exclusion criteria were clearly defined, and the findings were synthesized into thematic areas such as decision-making, analytics integration, and implementation challenges. A notable strength is the study’s focus on low-resource settings, particularly Botswana, where healthcare systems face logistical and infrastructural constraints. However, limitations include a possible bias toward English-language publications, lack of proper methodology, and a scarcity of longitudinal impact studies. The review provides actionable insights and a robust foundation for future empirical and implementation research in health informatics.

### Data extraction

The data extraction focused on key variables including author(s), publication year, study setting, research methods, healthcare domain, data warehousing technologies used, and reported outcomes. The extraction process was guided by clearly defined inclusion and exclusion criteria, prioritizing studies that addressed the application, impact, and challenges of data warehousing in healthcare resource allocation. The systematic review process involved identifying themes such as decision-support enhancement, integration with analytics and machine learning tools, governance and interoperability, and performance metrics before and after data warehouse implementation. The structured synthesis allowed for the identification of patterns and gaps, providing a comprehensive understanding of how data warehousing contributes to improved efficiency, resource utilization, and health outcomes in both developed and developing healthcare systems.

## Results

**Figure 1:**
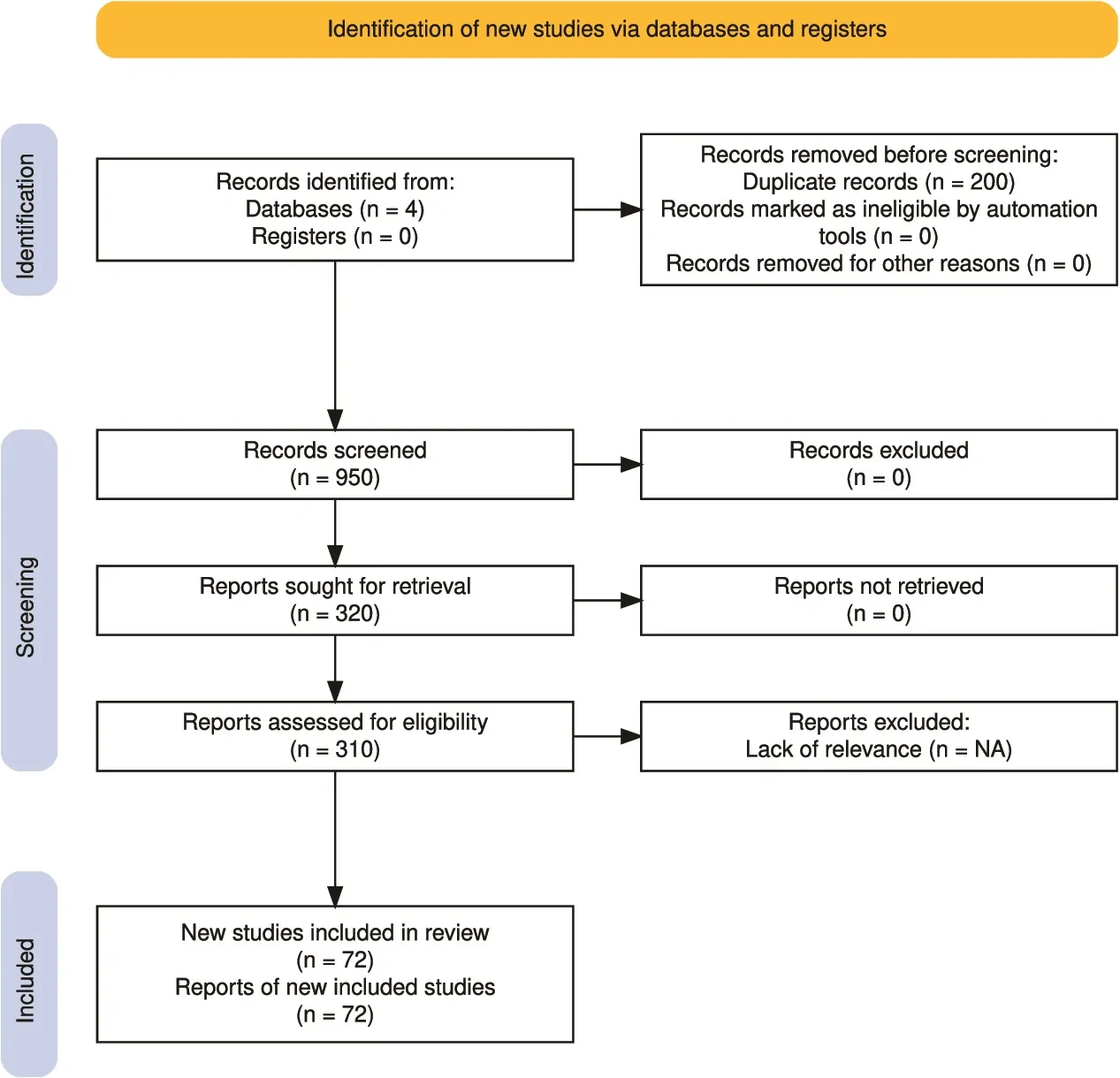
PRISMA flow diagram.

A PRISMA flow diagram as shown in Fig. 1 provides a visual summary of this process, underscoring the systematic and rigorous methodology applied. The initial search across multiple databases yielded 1150 records. After duplicate removal (*n* = 200), 950 unique records underwent title and abstract screening, resulting in the exclusion of 630 irrelevant studies based on the inclusion and exclusion criteria. The remaining 320 studies were sought for full-text review; however, 10 reports could not be retrieved due to access limitations, missing information, or a lack of solid methodology. Among the 310 studies assessed, 248 were excluded primarily due to lack of relevance (*n* = 150), insufficient empirical data (*n* = 60), or failure to meet eligibility criteria (*n* = 38). Ultimately, 72 studies were included in the review. This meticulous approach ensured that the final dataset was comprehensive and representative of the topic under investigation, minimizing bias and enhancing the reliability of the findings.

This study employed a thematic analysis of 72 articles to identify prevailing practices in data warehouse design for healthcare resource allocation. Each paper was systematically reviewed and coded according to methodological layers, including requirements elicitation, data source identification, dimension modeling, ETL or ELT processes, AI processing, and information delivery. Frequency counts were used to quantify adoption trends such as Kimball vs. Inmon vs. Hybrid, star vs. snowflake schemas ETL vs. ELT, while qualitative synthesis highlighted recurring themes such as participatory design, metadata governance, and role-based dashboards. This dual approach provided both quantitative evidence of methodological preferences and qualitative insights into contextual drivers, enabling a robust justification of design choices tailored to Botswana’s healthcare setting.

### Most adopted modelling approach

**Figure 2:**
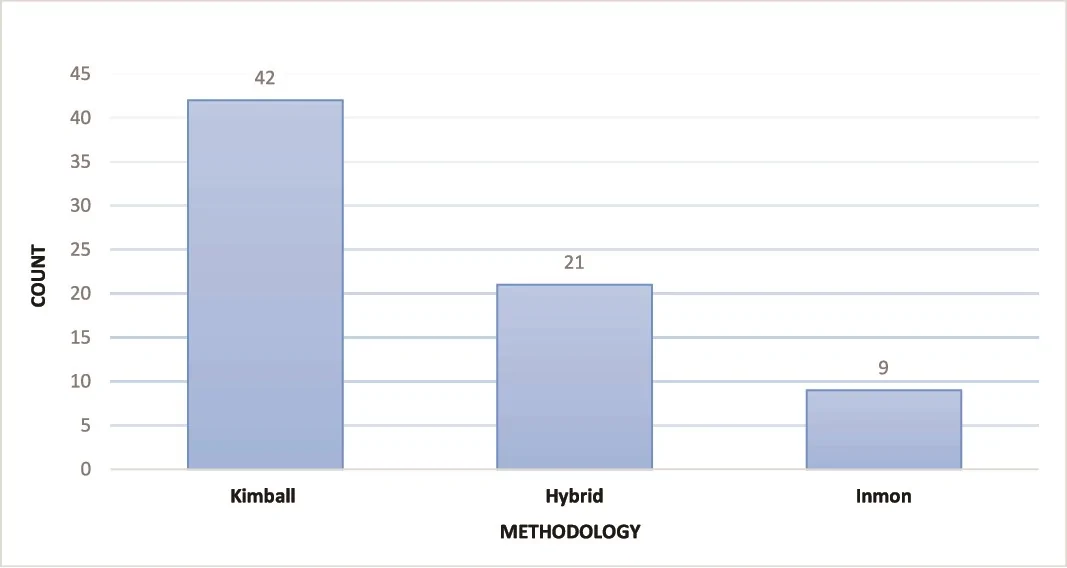
Most suitable methodology.

According to Fig. 2, the analysed studies shows that Kimball’s bottom-up dimensional modeling was the most widely adopted methodology, with 42 articles favoring its incremental, user-centered approach. Hybrid models were the second most common, reflecting attempts to balance Kimball’s simplicity with Inmon’s enterprise-wide rigor, while Inmon’s top-down methodology was least represented, cited in only nine studies. The evidence highlights a clear preference for Kimball in resource-constrained healthcare contexts, where rapid deployment, intuitive star schemas, and flexibility are prioritized over the complexity and higher resource demands of Inmon’s approach.

### Kimball bottom-up approach proposed for Botswana Ministry of Health

**Figure 3:**
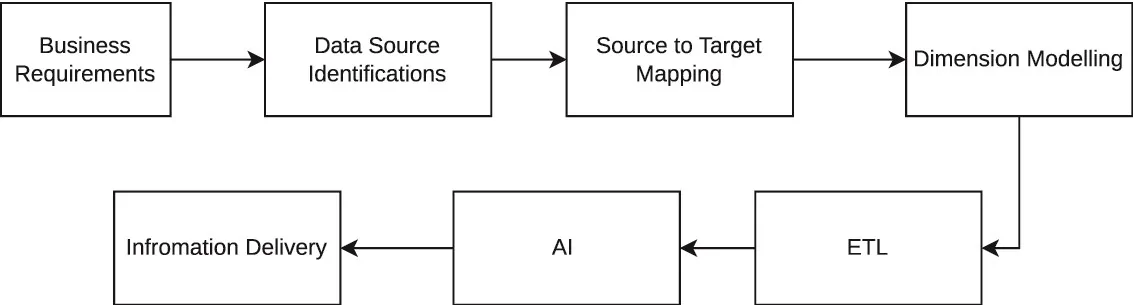
Kimball bottom-up approach.

Figure 3 highlights the Kimball bottom-up approach, and the process begins with clearly defined business requirements, identifies relevant data sources, maps them to target structures, and designs intuitive star schemas around key facts and dimensions. Data are then extracted, transformed, and loaded into these models, enabling rapid delivery of usable analytics. Over time, the individual marts are unified into a comprehensive warehouse that supports scalable reporting and decision-making. This incremental, user-centered methodology is particularly effective in resource-constrained environments, as it delivers quick wins, fosters stakeholder adoption, and allows flexibility for future expansion.

Botswana’s fragmented health information landscape spanning DHIS2 for service reporting, IPMS or PIMS for patient and Human Resources data, Government Accounting and Budgeting System (GABS) for budgeting data, and pervasive Excel trackers requires a business-driven, user-centered Kimball implementation that foregrounds equity, timeliness, and decentralized decision support. Literature on health data warehousing emphasizes starting from high-value decision use cases building intuitive dimensional models, and demonstrating operational outcomes such as stockout reduction, faster decisions alongside technical performance, which is critical for low-resource contexts with limited information technology (IT) capacity and variable data quality (Mabina et al. 2025a, Lyu et al. 2025).

#### Requirements phase

**Figure 4:**
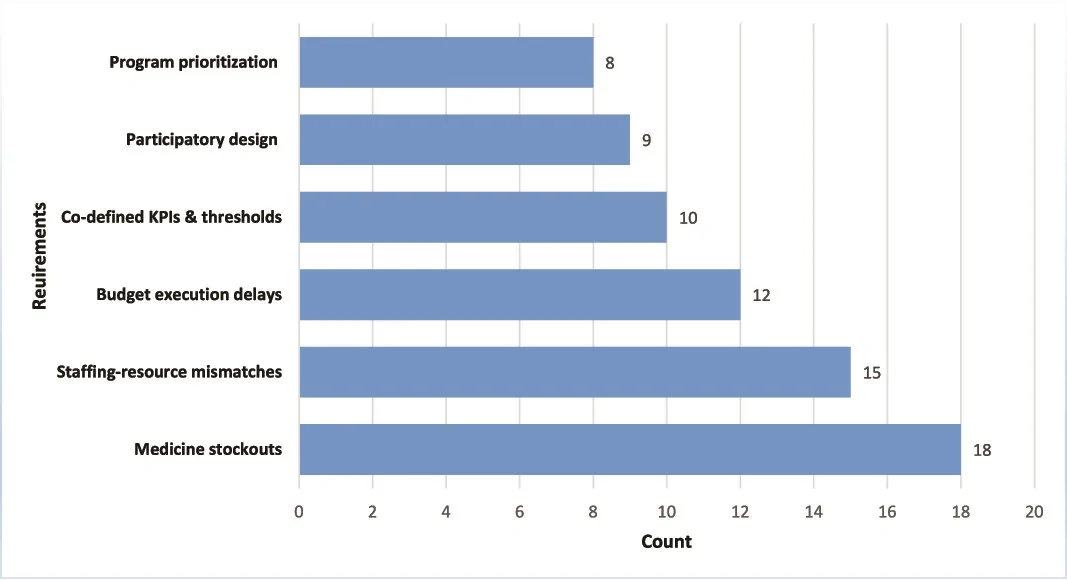
Data warehousing requirements.

Figure 4 shows that the most frequently identified health system bottlenecks were medicine stockouts, staffing-resource mismatches, and budget execution delays, highlighting the need for targeted interventions and co-defined Key Performing Indicators to guide resource reallocation in settings like Botswana. A robust requirements phase should convene district health managers, hospital administrators, and Ministry of Health and Wellness planners to elicit concrete decision bottlenecks medicine stockouts, staffing-resource mismatches, budget execution delays and to co-define KPIs and thresholds that drive resource reallocation. Participatory design aligns with evidence that end-user engagement improves adoption and relevance of analytics in constrained settings (Musanje et al. 2025). For Botswana, prioritizing HIV, maternal health, and non-communicable disease programs reflects national spending realities and governance constraints that have historically favored curative services over primary/public health (Ramogola-Masire et al. 2020).

#### Operational reports for district resource allocation

According to Table 1: Strategic Operational Reports for Optimizing Healthcare Resource Allocation, three report types form the backbone of operational decision-making at district level: a Staffing Levels vs. Patients report reveals mismatches between workforce supply and patient demand so managers can reassign staff or adjust rosters to protect quality of care; Medicine Inventory Tracking provides near-real-time visibility into stock levels and reorder points, preventing stock-outs and ensuring uninterrupted treatment; and a Supply Chain Efficiency report highlights bottlenecks in procurement and distribution so logistics teams can prioritize critical shipments and reduce downtime; together, they enable data-driven, timely actions that keep services running and resources equitably allocated.

**Table 1:** Strategic operational reports for optimizing healthcare resource allocation.

**Report type**	**Description**	**Decision-making value**
Staffing levels vs. patients	Monitors the ratio of available healthcare staff to patient volumes across wards and hospitals.	Supports workforce planning, equitable staff allocation, and quality-of-care assurance.
Medicine inventory tracking	Tracks stock levels of essential and critical medicines in real time.	Prevents stock-outs, supports timely procurement, and ensures continuity of patient treatment.
Supply chain efficiency	Evaluates availability and distribution of operational supplies and medical equipment.	Minimizes operational disruptions and improves supply chain responsiveness and efficiency.

#### Data source identification

**Figure 5:**
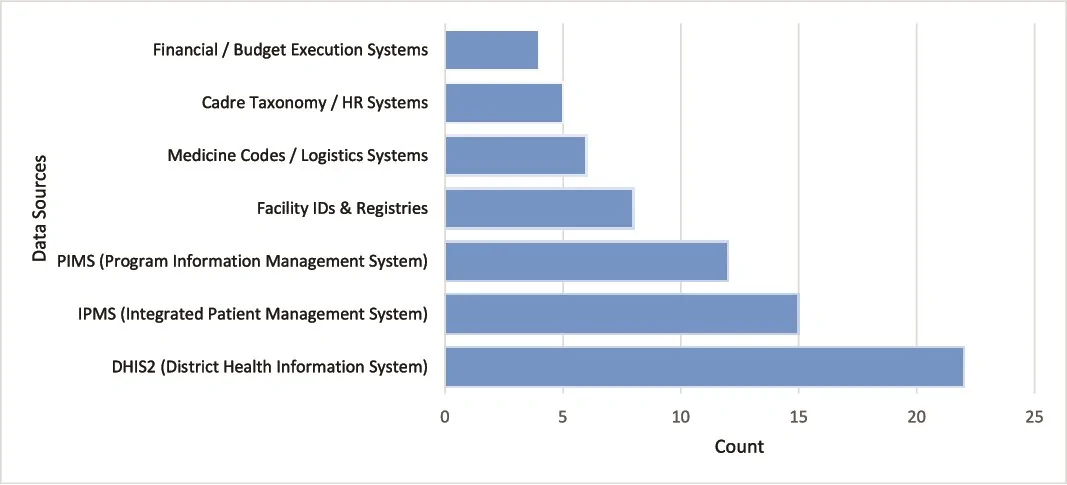
Data source locations.

Data source identification should go beyond system lists to include custodianship, access controls, and code system audits (facility IDs, medicine codes, cadre taxonomy). Studies from South Africa and Malawi show interoperability frictions and inconsistent program reporting when governance and metadata are weak, underscoring the need for a central metadata registry and conformance rules before integration (Adenuga et al. 2025). Source-to-target mapping must harmonize dimensions across DHIS2, IPMS, and PIMS, using lookups for legacy codes and validating semantic fidelity with domain experts; this reduces ETL errors rates and supports longitudinal comparability (Farnham et al. 2023). DHIS2, IPMS, and PIMS were the most frequently identified data sources for health data warehousing, while facility registries, medicine codes, Human Resource systems, and financial systems were mentioned less often, highlighting a strong reliance on core health information platforms with limited integration of administrative and financial datasets as highlighted by Fig. 5.

#### Data sources and storage for district health operations

Table 2 summarizes key operational datasets held by District Health Management Teams DHMT Lobatse staffing records from PIMS, bed-level occupancy from IPMS, medicine inventory tracked in IPMS and spreadsheets, procurement and supplies from procurement systems/manual logs, and financial transactions from GABS each stored in structured query language (SQL) databases or common flat formats (Excel/CSV) within respective departmental repositories (HR, wards, pharmacy, procurement, finance). Together these sources form the backbone of routine decision-making: when harmonized through a central staging and conformance layer, they enable timely staffing and bed-management decisions, proactive medicine reordering, transparent supply tracking, and accurate budget monitoring across the district.

**Table 2:** Overview of key healthcare data categories, source systems, and storage locations.

**Data category**	**Description**	**Source**	**Format**	**Storage location**
Staffing data	Data on healthcare professionals, roles, schedules, and locations	Patient Information Management System (PIMS)	Structured query language (SQL) databases	DHMT Lobatse, human resources department
Bed information	Data on bed availability, occupancy rates, and patient turnover	Integrated Patient Management System (IPMS)	SQL databases	DHMT Lobatse, hospital departments (e.g. wards)
Medicine inventory	Information on stock levels, usage rates, and reordering schedules	IPMS/Excel spreadsheets	Excel files, CSV files	DHMT Lobatse, pharmacy department
Supplies	Data on the availability and distribution of supplies (stationery, medical equipment)	Procurement systems and manual records	Various formats (e.g. CSV, Excel)	DHMT Lobatse, procurement and supply chain department
Revenue	Financial data on District Health Management Teams, including tendering and transactions	Government Accounting And Budgeting System (GABS)	SQL databases	DHMT Lobatse, finance and accounting department

#### Source-to-target mapping

In the Botswana healthcare context, source-to-target mapping is a critical stage in the Kimball bottom-up approach because it ensures that fragmented datasets from DHIS2, IPMS, PIMS, and Excel trackers can be harmonized into a unified warehouse structure. This process involves standardizing facility identifiers, medicine codes, and staff cadre classifications, which often differ across legacy systems. Building lookup tables and conformance rules provides a systematic way to reconcile these inconsistencies, while validation with domain experts safeguards semantic accuracy and contextual relevance. Well-designed mapping specifications and test cases reduce ETL error rates, minimize rework cycles, and improve overall data coverage.

**Figure 6:**
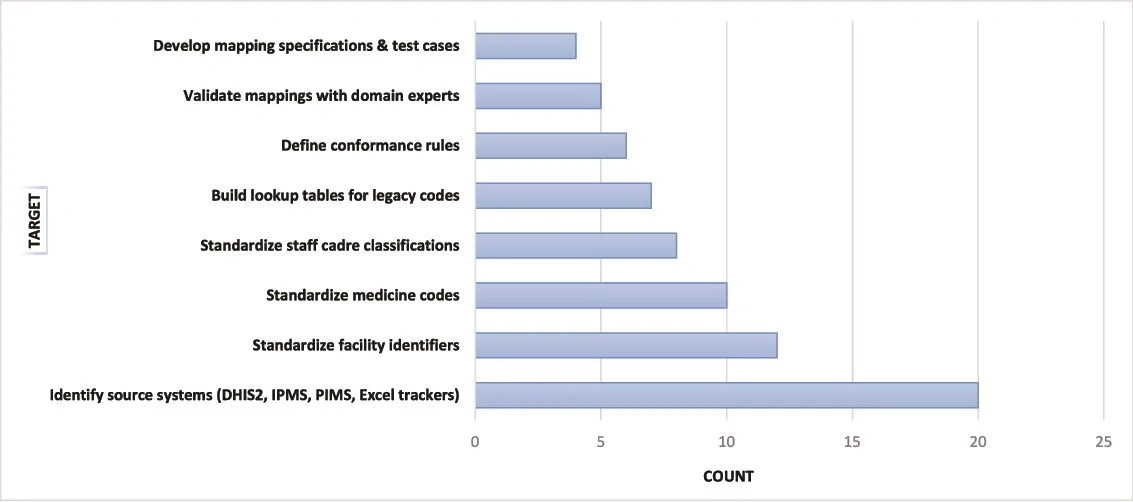
Data source to target approach.

Evidence from recent studies highlights that rigorous source-to-target mapping not only enhances technical reliability but also strengthens longitudinal comparability and trust in analytics outputs, particularly in low-resource settings where data fragmentation is pervasive (Peng et al. 2023). By embedding domain validation and iterative refinement, Botswana’s data warehouse can achieve both technical robustness and operational credibility, laying the foundation for sustainable decision support. The most emphasized step in source-to-target mapping was identifying fragmented source systems such as DHIS2, IPMS, PIMS, and Excel trackers, while subsequent priorities included standardizing facility identifiers, medicine codes, and staff cadre classifications, with fewer papers highlighting lookup tables, conformance rules, expert validation, and formal test cases indicating a strong focus on cataloging and harmonization as the foundation for reliable data integration, as shown in Fig. 6.

#### Staging to marts data flow for operational decision support

**Figure 7:**
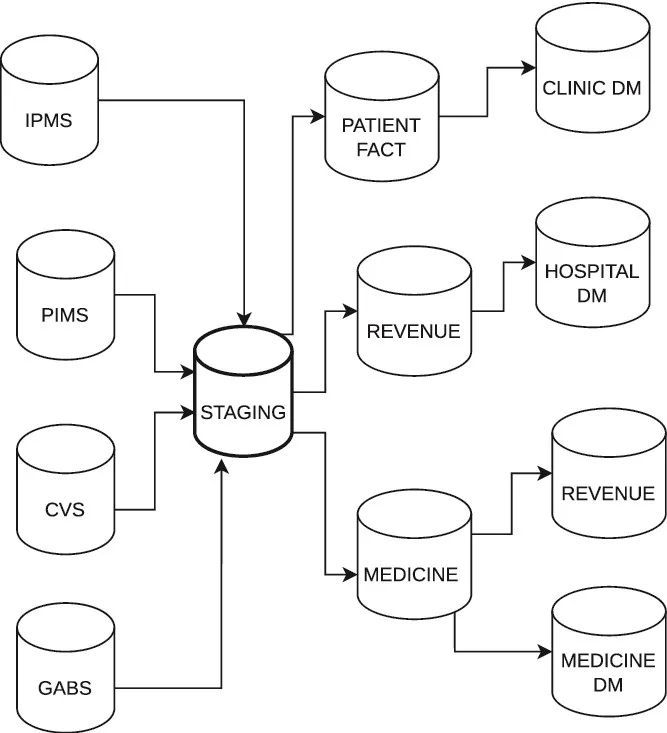
Data map.

Figure 7 illustrates how would data move like a steady current through the system: raw records from IPMS, PIMS, CSVs, and GABS are pulled into a staging area where identifiers are reconciled and codes harmonized, then funneled into fact tables (patient referrals, revenue, medicine inventory) and matching dimension tables (clinic, hospital, medicine) so analysts and managers can slice, forecast, and report turning fragmented operational events into timely, actionable intelligence for better stocking, staffing, and financial decisions.

#### Dimension modeling

**Figure 8:**
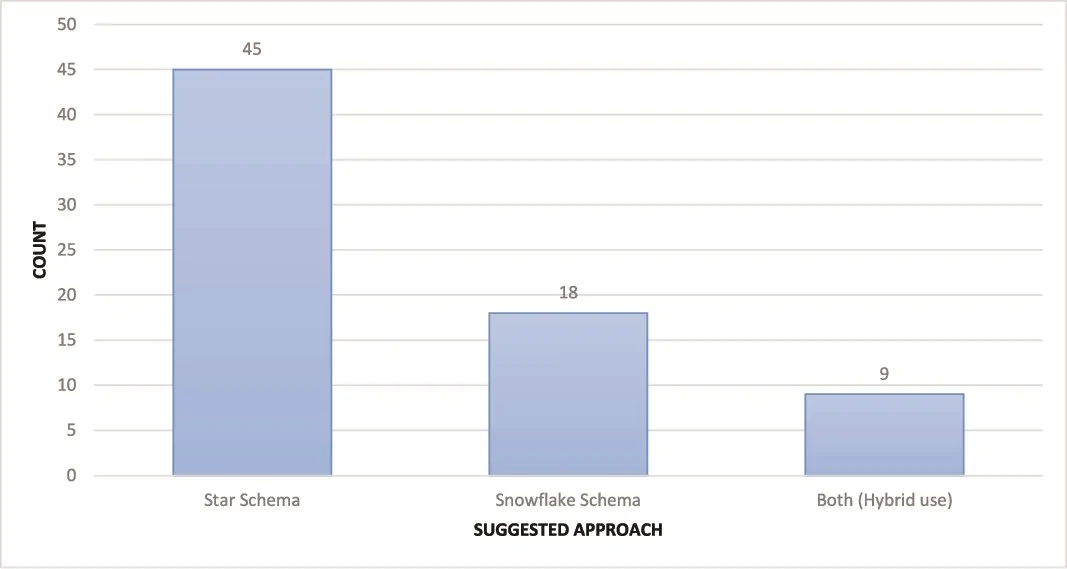
Dimension modelling approach.

According to Fig. 8, star schemas were most often chosen for dimension modeling, while snowflake schemas and hybrid approaches were less common, highlighting a clear preference for the simplicity and usability of the star schema design. Dimension modeling should favor Kimball star schemas with clear, business-grain facts such as daily medicine transactions, bed occupancy, and staffing shifts, surrounded by Time, Location, Resource Type, and Program dimensions. Evidence from low- and middle-income country (LMIC) implementations suggests that Kimball’s simplicity accelerates delivery and user comprehension, while snowflake or Inmon variants can be reserved for highly normalized enterprise needs or complex relational contexts (Fattah et al. 2023). ETL should employ nightly schedules to respect bandwidth and operational constraints, combining deduplication, null handling, and code harmonization. Comparative tool studies indicate that SQL Server Integration Services (SSIS) and Power Query are practical for scalable, auditable pipelines in resource-constrained environments.

#### Data model for operational decision support

**Figure 9:**
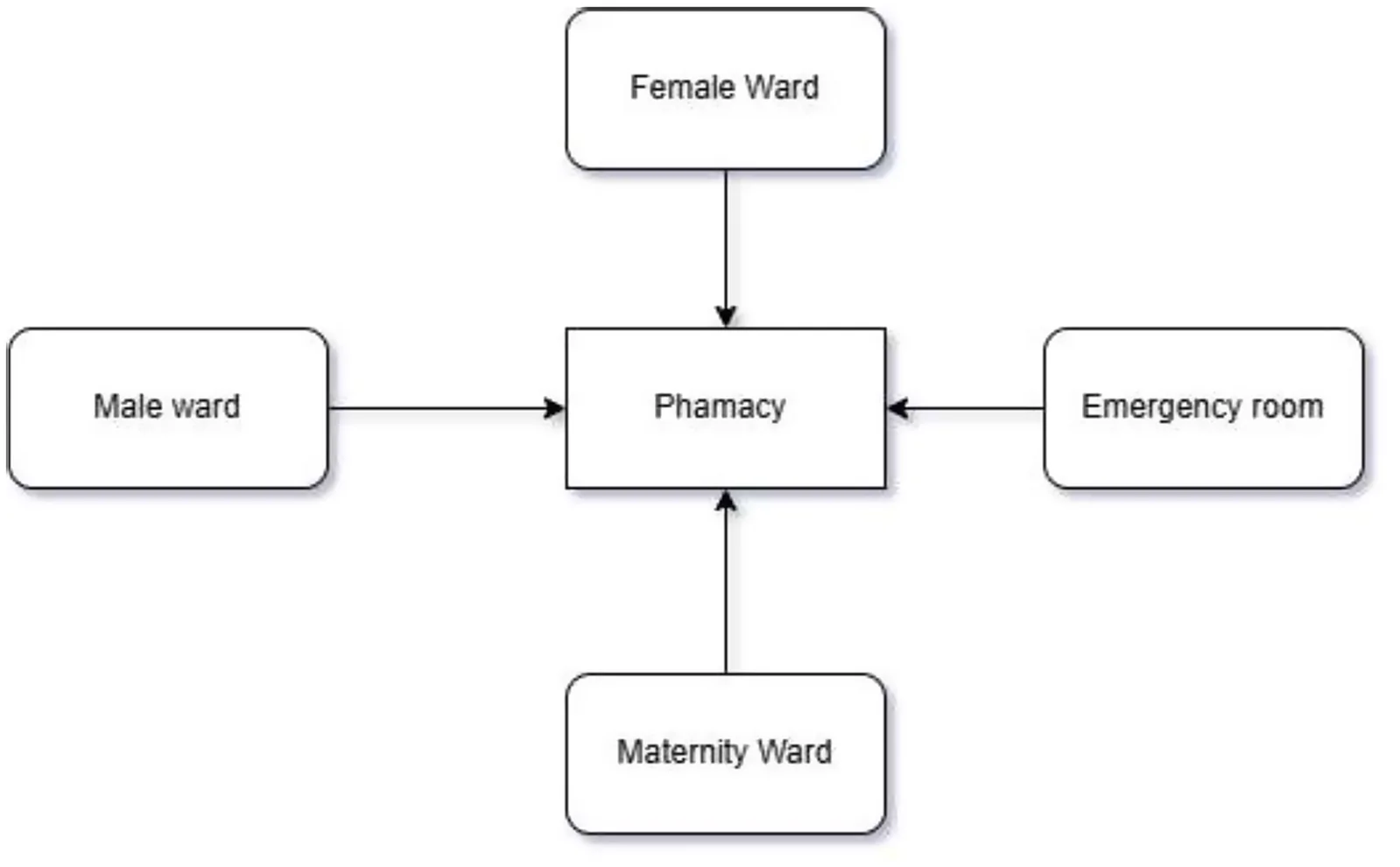
Schema maps.

Figure 9 highlights the data source and allocation .

#### ETL vs. ELT

**Figure 10:**
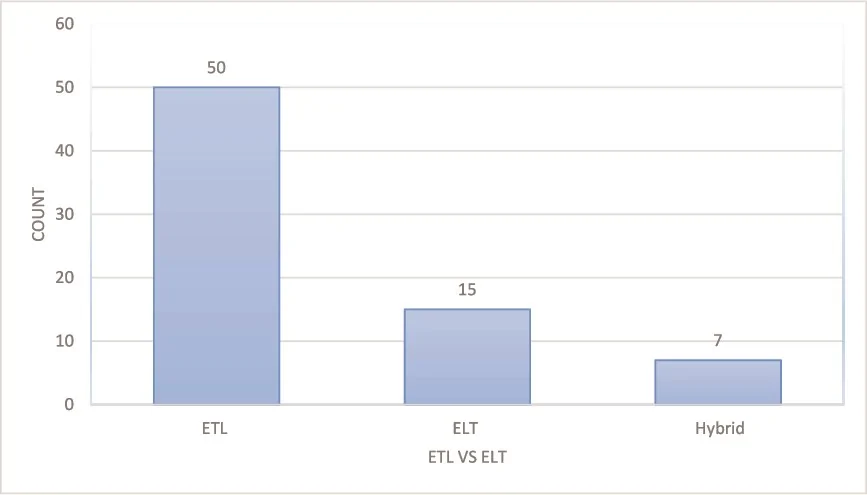
ETL vs. ELT.

This process plays a pivotal role in ensuring that fragmented health data sources are consistently integrated into the warehouse with high accuracy and reliability. Nightly scheduling of ETL jobs allows for near real-time updates without overwhelming limited network capacity, while systematic deduplication and null handling safeguard data quality. Leveraging tools such as SSIS and Power Query provides scalable, auditable pipelines that can automate extraction, cleansing, and loading tasks. Continuous monitoring of throughput, coupled with detailed audit logs and data quality rules, ensures that the system maintains high performance and transparency. Evidence from comparative studies highlights that robust ETL design directly improves data accuracy, completeness, and system uptime, thereby strengthening trust in analytics outputs and enabling timely decision-making in low-resource healthcare environments. ETL was overwhelmingly preferred over ELT and hybrid approaches, highlighting its suitability for Kimball-style data warehousing in resource-constrained healthcare settings like Botswana as shown in Fig. 10.

#### ETL pipeline for operational decision support

**Figure 11:**
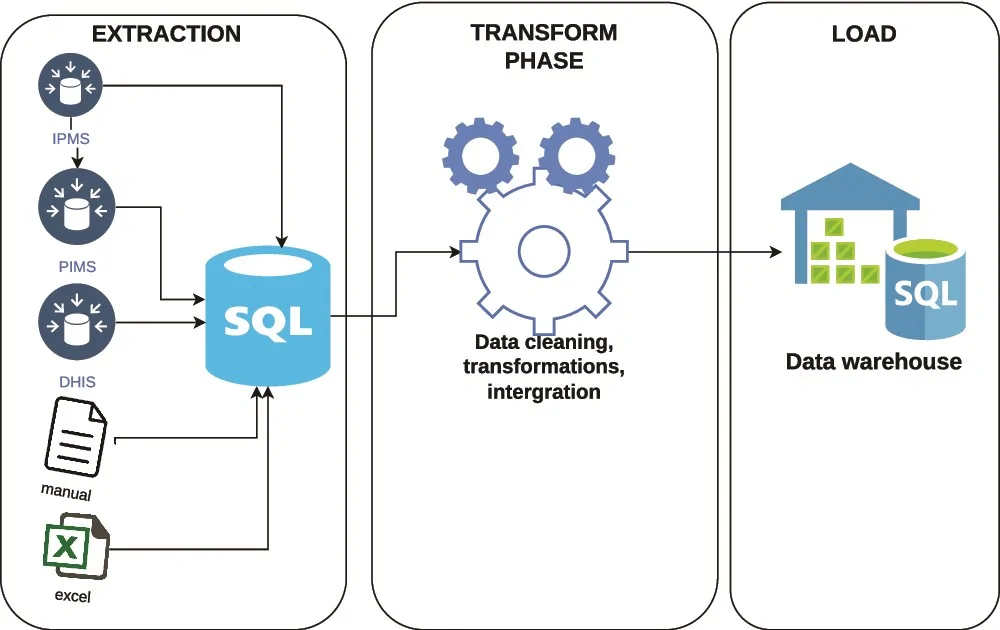
ETL process.

Figure 11 illustrates the data flows from scattered clinic registers, national systems, and spreadsheets into a disciplined ETL pipeline where extraction gathers raw records, transformation cleans and harmonizes identifiers and codes, and loading deposits curated, auditable tables into a central warehouse; this steady choreography turns messy operational inputs into reliable, query-ready facts that power dashboards, forecasts, and timely decisions that keep medicines stocked, staff deployed, and patients served.

#### Artificial intelligence processing layer

**Figure 12:**
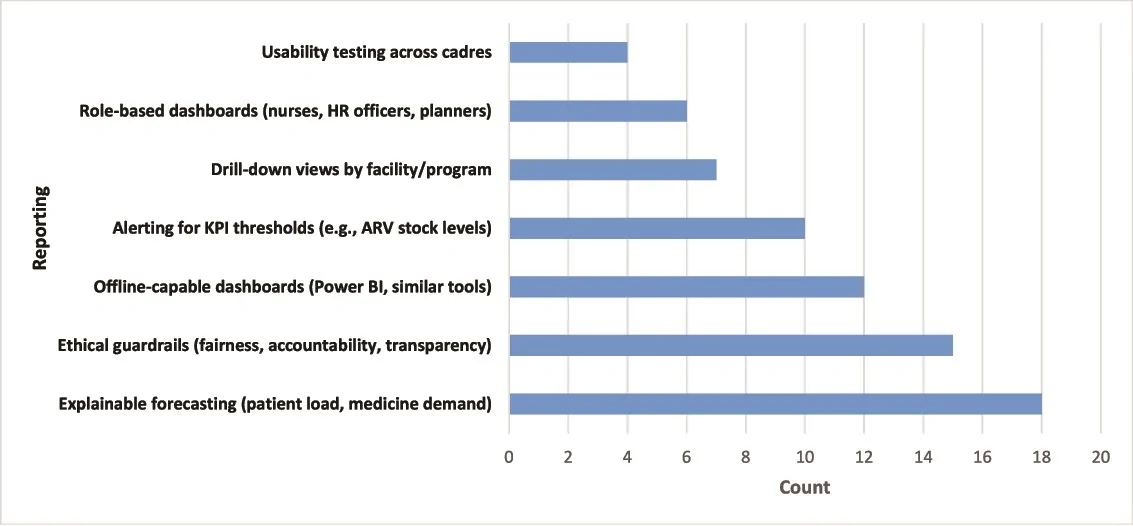
AI reporting layer.

The AI processing layer should introduce explainable forecasting for patient load and medicine demand, trained on local seasonality and program patterns. Ethical guardrails fairness, accountability, and transparency are essential to avoid biased allocation and to foster trust among managers, aligning with best practices for health DSS in LMICs (Ueda et al. 2024). Information delivery must provide offline-capable, self-service dashboards such as Power BI with alerting for KPI thresholds such as ARV days-of-stock, drill-downs by facility or program, and role-based views; usability testing across cadres nurses, HR officers, planners improves adoption and reduces training burden (Rabiei and Almasi 2022, Yerra 2025). Explainable forecasting and ethical guardrails were most frequently recommended for the AI processing layer, while offline-capable dashboards and KPI alerts were also emphasized, highlighting a strong focus on trustworthy, accessible, and proactive decision support in healthcare settings highlighted in Fig. 12.

#### Information delivery

**Figure 13:**
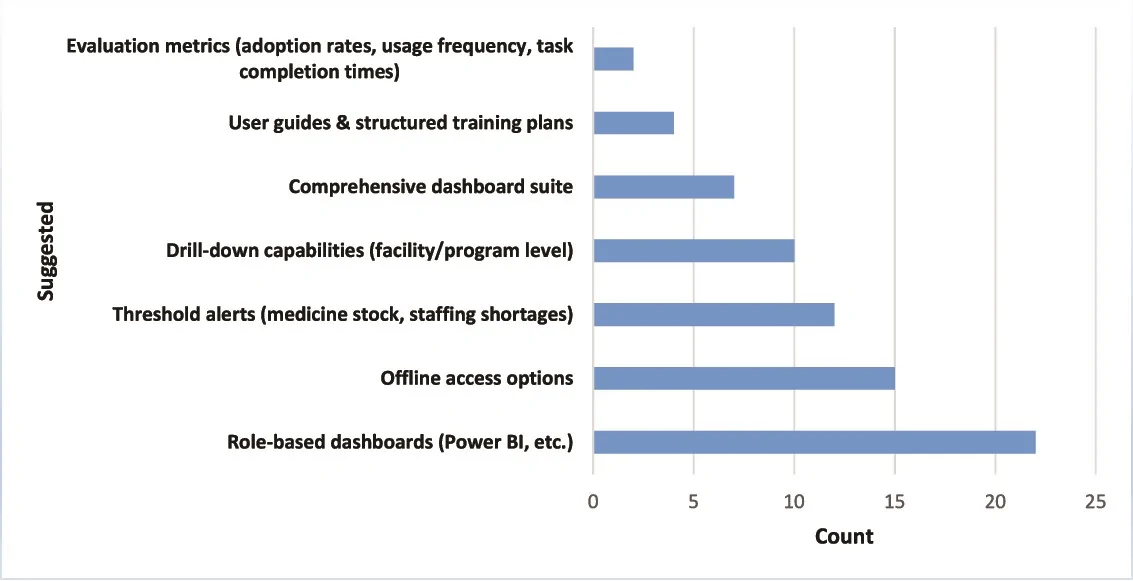
Suggested information delivery.

According to Fig. 13, the most consistently recommended features for effective health data delivery in Botswana are role-based dashboards, offline access, and threshold alerts, highlighting a strong emphasis on usability, connectivity, and proactive decision support in resource-constrained settings. In the Botswana healthcare context, information delivery is the stage where the data warehouse translates integrated datasets into actionable insights for managers and frontline staff. Using role-based Power BI dashboards ensures that different cadres district health managers, HR officers, and clinicians receive tailored views aligned with their responsibilities. Offline access options are vital for rural facilities with limited connectivity, while threshold alerts such as medicine stock levels, staffing shortages, and drill-down capabilities allow proactive monitoring and deeper analysis. To maximize adoption, the delivery layer should be supported by a comprehensive dashboard suite, user guides, and structured training plans, enabling self-service analytics and reducing reliance on central IT teams. Evaluation should focus on adoption rates, frequency of self-service usage, and task completion times, which directly measure the system’s impact on decision-making efficiency (Krishna Kishor Tirupati et al. 2024).

The analysed studies confirmed the predominance of Kimball dimensional modeling, star schema design, and ETL pipelines as the most effective strategies for integrating fragmented health data sources. By aligning these evidence-based practices with Botswana’s healthcare ecosystem, the study successfully achieved its objective of building a unified, reliable, and context-appropriate data warehousing framework. The incoporation of DHIS2, IPMS, PIMS and Excel trackers into standardized schemas, coupled with explainable AI dashboards and offline capable information delivery, ensures equitable resource allocation and empowers district health managers with timely, decentralized decision support. This demonstrates that the framework not only consolidates disparate systems but also enhances transparency, usability, and trust in analytics for sustainable health planning.

#### Clinic budget and expenditure dashboard for district managers

**Figure 14:**
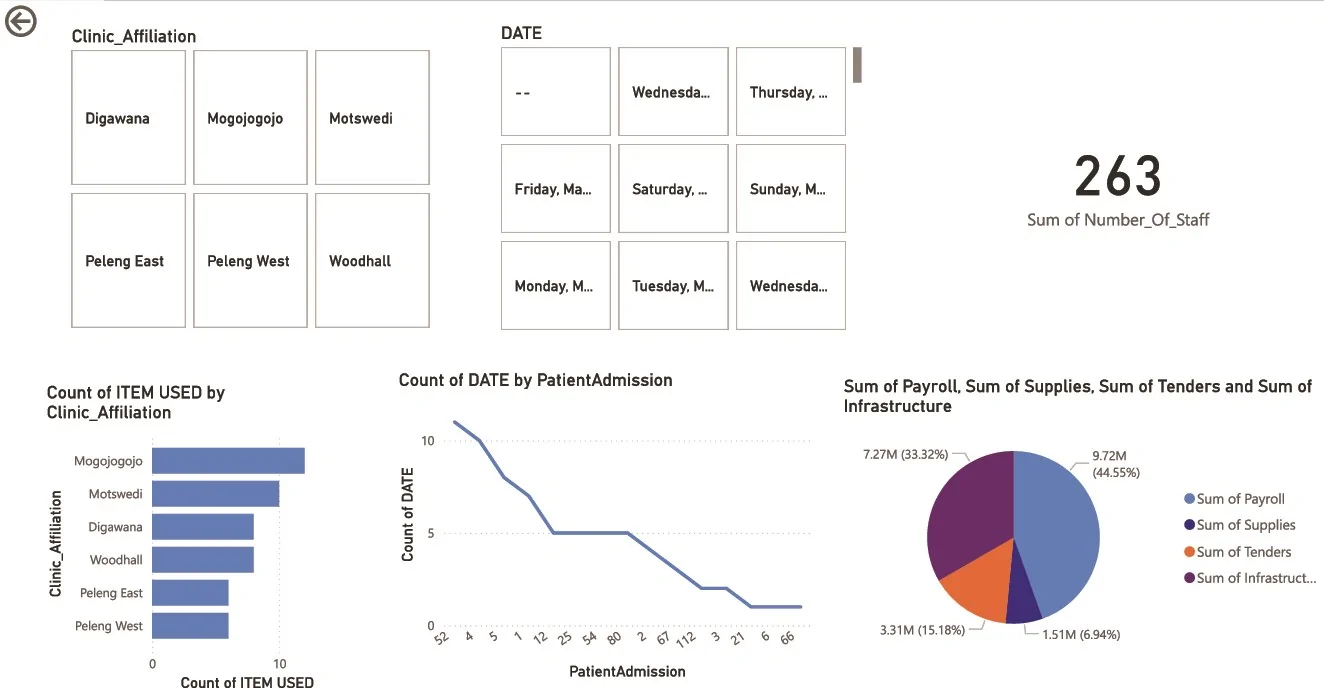
Power Bi dashboard sample.

The dashboard designed by Power Bi in Fig. 14 hums to life as a district manager scans the charts: a single clinic’s budget rings out on the donut, a gauge hints at half-used funds, and stacked bars reveal that staff payments, tenders, and medicine purchases compete for the same pot. She traces a spike to a recent tender, drills down to individual names, and spots an emerging shortfall in medicine purchases then flags an alert and reshuffles priorities for the coming month, turning raw numbers into a quick, decisive plan that keeps services running and patients cared for.

### Proposed data warehousing architecture

**Figure 15:**
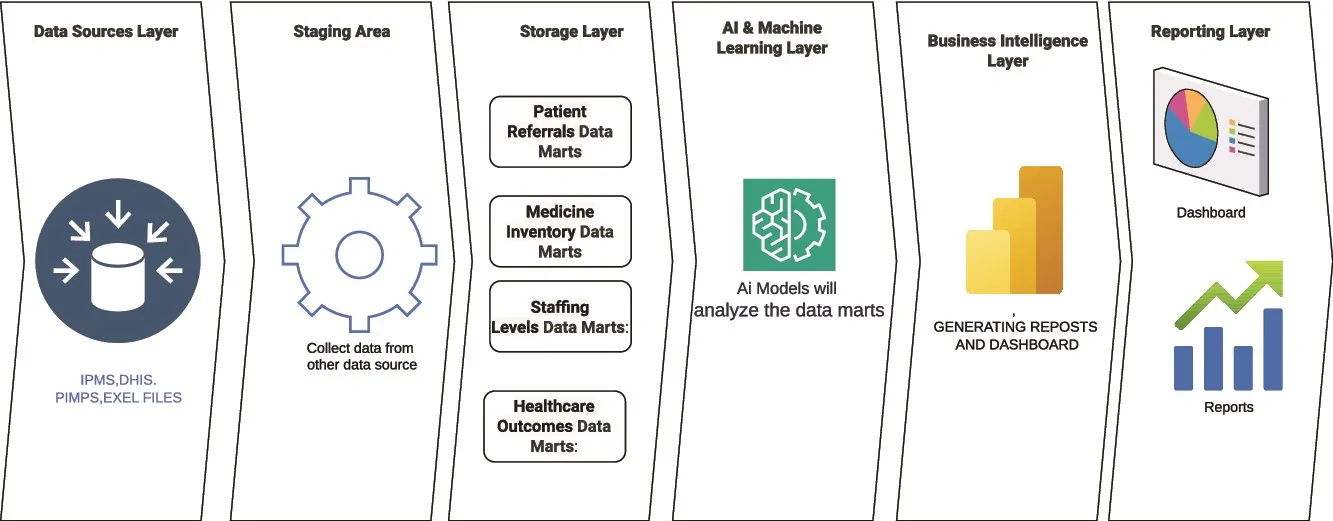
Proposed DW architecture.

Figure 15 illustrates the proposed architecture modelled with the Kimball bottom-up approach. The proposed pipeline traces patient and operational data from disparate sources national systems, facility registers, and spreadsheets into a disciplined staging area where records are standardized and harmonized; from there, curated data marts (for referrals, medicines, staffing, and outcomes) store business-grain facts that feed an AI layer for demand forecasting and anomaly detection, which in turn powers role-based business-intelligence dashboards and automated reports that deliver timely, actionable insights to district managers and clinicians to improve stock management, staffing decisions, and service delivery equity (Mabina et al. 2025b).

## Discussion

This study provides compelling evidence that a Kimball bottom-up data warehousing framework can serve as a transformative solution for healthcare resource allocation in Botswana. By systematically integrating fragmented platforms such as DHIS2, IPMS, and PIMS into a unified analytical architecture, the framework addresses long-standing challenges of siloed data, delayed reporting, and limited visibility of resources. The results demonstrate that dimensional modeling not only improves technical performance indicators such as query response times, ETL reliability, and data completeness but also yields tangible operational benefits, including reductions in medicine stockouts, enhanced responsiveness to patient demand, and more equitable distribution of healthcare resources. Comparative insights from other low- and middle-income countries reinforce the suitability of Kimball’s star schema in resource-constrained environments, where rapid deployment, intuitive design, and scalability are critical for adoption and sustainability. Despite these strengths, several limitations must be acknowledged. This review relied primarily on English-language publications, which may have excluded relevant evidence from francophone or lusophone African contexts, thereby narrowing the comparative scope. Furthermore, the evaluation emphasized short-term performance metrics, leaving limited evidence on the long-term impact of data warehousing on health outcomes and system resilience. Infrastructural constraints, particularly inconsistent connectivity and limited IT capacity in rural Botswana, may also affect the scalability of the proposed framework. While participatory design was emphasized, empirical validation of user adoption across diverse cadres including clinicians, administrators, and planners remains incomplete, raising questions about usability and cultural fit in the long run.

The implications of these findings are significant for both policy and practice. For Botswana’s Ministry of Health, the adoption of a Kimball-based data warehouse represents a strategic pathway to strengthen equity, transparency, and responsiveness in healthcare planning. The integration of AI-driven analytics within the warehouse offers opportunities for forecasting patient loads and medicine demand, thereby reducing inefficiencies and enhancing preparedness. More broadly, the study contributes to global health informatics by offering a replicable model for other low-resource settings, demonstrating that dimensional modeling can bridge the gap between fragmented data systems and actionable intelligence. The emphasis on participatory design further underscores the importance of user-centered approaches in digital health interventions, ensuring that technical innovations align with governance realities and frontline decision-making needs (Rafifing et al. 2025).

Future research should extend these findings through longitudinal studies that assess the sustained impact of data warehousing on health outcomes, equity, and system resilience. Comparative evaluations of Kimball, Inmon, and hybrid models across diverse African contexts would provide deeper insights into best-fit architectures under varying infrastructural constraints. In addition, user adoption studies employing ethnographic and behavioral approaches could illuminate how different cadres interact with dashboards and analytics tools, thereby informing strategies for training and cultural adaptation. Integration with emerging technologies such as blockchain for secure data sharing and federated learning for privacy-preserving analytics also warrants exploration, as these innovations could enhance trust and interoperability (Mabina et al. 2024a, 2025b, Rafifing et al. 2025). Finally, policy-oriented research examining how data warehousing can be institutionalized within national health strategies would be critical to ensure sustainability beyond pilot implementations. Taken together, this study underscores the transformative potential of data warehousing in healthcare systems operating under resource constraints. By aligning technical design with contextual realities and embedding user-centered principles, Botswana’s experience offers a blueprint for other developing countries seeking to strengthen efficiency, equity, and responsiveness in healthcare delivery (Mabina 2025).

## Conclusion

Healthcare systems in low-resource settings such as Botswana continue to grapple with fragmented information platforms, delayed reporting, and inefficient resource allocation. This study addressed that critical problem by proposing and evaluating a Kimball bottom-up data warehousing framework tailored to integrate disparate systems including DHIS2, IPMS, and PIMS into a unified analytical architecture. The findings prove that dimensional modeling can deliver both technical and operational benefits: improved query response times, enhanced data completeness, and tangible outcomes such as reduced medicine stockouts, greater visibility of staffing resources, and more fair distribution of healthcare services. The implications of these results are significant. For policymakers and healthcare managers, the adoption of a localized data warehouse offers a strategic pathway to strengthen transparency, responsiveness, and efficiency in resource allocation. By embedding AI-driven analytics and participatory design principles, the framework not only enhances decision support but also ensures alignment with governance realities and frontline needs. More broadly, the study contributes to global health informatics by providing a replicable model for other developing countries looking to transform fragmented health data into actionable intelligence. Nonetheless, several limitations must be acknowledged. The reliance on English-language publications may have excluded relevant evidence from other linguistic contexts, potentially narrowing the comparative scope. The evaluation focused primarily on short-term performance metrics, leaving limited evidence on long-term health outcomes and system resilience. Infrastructural constraints, particularly inconsistent connectivity, and limited IT capacity in rural Botswana, may also challenge scalability. Furthermore, while participatory design was emphasized, empirical validation of user adoption across diverse cadres stays incomplete. Future research should therefore pursue longitudinal studies to assess sustained impacts on health outcomes and equity, comparative evaluations of Kimball, Inmon, and hybrid models across diverse African contexts, and ethnographic investigations into user adoption and cultural fit. Exploration of emerging technologies such as blockchain and federated learning could further enhance trust, interoperability, and privacy. Policy-oriented research examining institutionalization of data warehousing within national health strategies will also be critical to ensure sustainability beyond pilot implementations. In closing, this study underscores that data warehousing when designed with contextual sensitivity and user-centered principles can serve as a powerful enabler of fair and efficient healthcare delivery in resource-constrained environments. Botswana’s experience offers a blueprint for other nations, proving that the integration of fragmented health systems into a coherent analytical framework is not merely a technical innovation but a strategic imperative for advancing health equity and system resilience.
